# 2412 Cost effectiveness analysis of operative Versus antibiotic management for uncomplicated appendicitis

**DOI:** 10.1017/cts.2018.279

**Published:** 2018-11-21

**Authors:** Eric Stulberg, Alexander Zheutlin, Raymond Strobel, Katherine He, Adelyn Beil

**Affiliations:** 1 Northwestern University; 2 University of Michigan School of Medicine

## Abstract

OBJECTIVES/SPECIFIC AIMS: (1) Evaluate the relative incremental cost-effectiveness [cost per quality-adjusted life year (QALY) gained] of antibiotics, laparotomy, and laparascopy for the initial treatment of uncomplicated appendicitis. (2) Detect if the relative incremental cost-effectiveness of each treatment differs by age, namely in pediatric patients, adult patients, and geriatric patients. (3) Use deterministic and probabilistic sensitivity analyses to assess the robustness of our findings when varying multiple model parameters. METHODS/STUDY POPULATION: Study Population and Analytic Approach: The population under analysis is a simulated population of those aged 1–90 diagnosed with uncomplicated appendicitis with computed tomography (CT) in the emergency department. Pregnant women and those younger than 1 year old were excluded from our analysis. We simulated our population through a Markov state-transition simulation model. Using this model, we estimated the lifelong costs and effects on QALYs from the use of antibiotics, laparoscopy, and laparotomy for a given hypothetical individual with uncomplicated appendicitis. This model allowed for the incorporation of both the short-term and long-term effects of each respective treatment option. The primary outcome of the model was the cost per additional QALY gained. The analysis was conducted using a healthcare perspective. A 100 age-year time horizon was used. A 3% discount rate was applied to both the costs and effects in the model. Transition states are depicted. Surgical state rates were derived from HCUP. Treatment failure of antibiotics was defined as recurrent appendicitis within one year of antibiotic treatment. This was determined using results from prior RCTs and a Cochrane review of antibiotic management for uncomplicated appendicitis. Recurrent appendicitis was defined as recurrent appendicitis after 1 year of antibiotic treatment, using rates of appendicitis applied to the general population by age group. National age-adjusted mortality rates were applied to account for death due to causes unrelated to appendicitis. To assess differential results by age, different acute and long-term outcome, cost, and state transition rates were applied to 3 age groups: a pediatric group (1–17 years old), an adult group (18–64 years old), and a geriatric group (65+ years old). As an individual progressed through the model until age 100, the respective parameters would change to adjust for the transitions between the 3 life stages. Outcomes After Appendicitis: Lifetime QALYs were incorporated throughout the study for short-term and long-term health states. There is limited availability of QALY data in the literature pertaining to the health states specific to appendicitis. Due to this limitation, however, calculated quality of life (QoL) indices for 2015 created by Wu *et al*. were utilized for this study. QALYs were subsequently derived by multiplying QoL by the appropriate duration of time spent in a respective health status. Transition rates between health states were abstracted from the existing literature. Costs: Direct medical costs were obtained from HCUP statistics from the 2014 fiscal year for all age groups in the nationwide network. This database contains all costs of care related to surgical appendicitis intervention, however it lacks costs associated with antibiotic-only management. To account for these costs, data was extracted from current available literature, and the resulting average was applied to our model. Sensitivity Analysis: One-way analyses by cost of procedure and effectiveness of antibiotic protocol were undertaken to account for regional variation in costs and improvements in antibiotic therapy, respectively. For cost of procedure sensitivity analysis, costs were varied by 1 standard deviation below and above the mean cost per treatment group per age. These costs were then compared to a designated reference group. Antibiotic sensitivity analysis was conducted by reducing the effectiveness of antibiotics from the maximum reported effectiveness down to 0, with the goal of obtaining a level of effectiveness at which antibiotics were no longer cost-effective. A probabilistic Monte-Carlo sensitivity analysis was then employed to determine the percent likelihood of each treatment arm being cost-effective at a level of $100,000 per additional QALY. The probabilistic sensitivity analysis was then repeated to determine the percent likelihood of each treatment arm being the dominant option, in that it lowers costs and adds QALYs. RESULTS/ANTICIPATED RESULTS: Our model examined the cost-effectiveness of 3 different treatment options for patients with acute uncomplicated appendicitis: laparoscopic appendectomy, laparotomy appendectomy, and an antibiotic regimen. We first examined the cost-effectiveness of each of these strategies in comparison to laparotomy. Laparoscopic appendectomy was shown to be superior to laparotomy in regards to costs and QALYs for patients ages 18 to 65+, while there was very little difference for patients ages 1–17. For those aged 1–17, laparoscopy had an additional cost of $90.00 with an associated gain of 0.1 QALYs compared with laparotomy. For those aged 18–64, laparoscopy had a net cost-savings of $3437.03 with an associated gain of 0.13 QALYs compared with laparotomy. For those aged 65+, laparoscopy had a net cost-savings of $5713.55 with an associated gain of 0.13 QALYs compared to laparotomy. Antibiotic management was superior to laparotomy as it relates to both costs and QALYs for all 3 age cohorts. For those aged 1–17, antibiotic management had a net cost-savings of $5972.55, with an associated gain of 0.6 QALYs compared with laparotomy. For those aged 18–64, antibiotic management had a net cost-savings of $6621.00 with an associated gain of 0.5 QALYs compared with laparotomy. For those aged 65+, antibiotic management had a net cost-savings of $11,953.00 with an associated gain of 0.21 QALYs compared with laparotomy. We then assessed the cost-effectiveness of antibiotics relative to laparoscopy. In all 3 age groups, antibiotics added QALYs and were cost-saving. For those aged 1–17, antibiotic management had a net cost-savings of $6062.55, with an associated gain of 0.6 QALYs compared with laparotomy. For those aged 18–64, antibiotic management had a net cost-savings of $3183.97 with an associated gain of 0.5 QALYs compared with laparotomy. For those aged 65+, antibiotic management had a net cost-savings of $6239.45 with an associated gain of 0.21 QALYs compared with laparotomy. Sensitivity Analysis: We first examined the effect of varying costs on our results. Costs for all interventions were varied by 1 standard deviation above and below the average costs used in our original model, yielding 3 cost estimate levels: high cost (1 standard deviation above), middle cost (average cost reported in model), low cost (1 standard deviation below). For all 3 cost estimate levels of antibiotics, antibiotics persistently dominated laparotomy for all 3 age groups. Laparoscopy dominated at all cost levels in age groups 18–64 and 65+ but had a positive ICER for both high and medium cost levels in the 1–17 age group. We then varied effectiveness (one minus the failure rate) of antibiotic treatment in each age group to assess at what level of effectiveness to antibiotics become dominant relative to laparotomy. In ages 1–17, antibiotic treatment became dominant at 43.8%; in ages 18–64, antibiotic treatment became dominant at 33%; and in ages 65+, there was no level of antibiotic effectiveness that did not result in this therapy being dominant over laparotomy. Probabilistic Monte-Carlo sensitivity analysis is pending, but we anticipate antibiotics having a high likelihood of being both cost-effective and dominant relative to the other 2 treatment options. DISCUSSION/SIGNIFICANCE OF IMPACT: We performed a cost-effective analysis comparing surgery versus antibiotic management for uncomplicated appendicitis. Our study found that antibiotic therapy was the dominant strategy in all age groups as it yielded lower costs and additional QALYs gained compared with laparotomy and laparoscopy. Appendicitis is the most common surgical emergencies worldwide, with a lifetime risk of 6.9% in females and 8.6% in males (Körner 1997). For over 100 years, open appendectomy had been the established treatment for appendicitis, but current management has evolved with the advent of laparoscopy and now growing use of antibiotics for treatment of appendicitis. There is growing interest in nonoperative management of uncomplicated appendicitis, given both an aging population that is increasingly frail and vulnerable to surgical complications and concerns over skyrocketing medical costs. Our model showed that antibiotic-only management was cost-effective in all age groups. This has important implications for management of appendicitis, where current management is to offer antibiotic-only management only in the “rare cases” where the patient is unfit for surgery or refuses surgery. Our data show that medical management of appendicitis not only is cheaper, but also provides more QALYs in all age groups. Our study has several limitations. First, we conducted our analysis under the assumption that all patients will be cured of appendicitis following surgical intervention. Some patients following appendectomy will develop symptoms of appendicitis and be diagnosed with “stump appendicitis,” which can occur in stumps as short as 0.5cm and can present as late as 50 years following initial surgery (Kanona, 2012). Additionally, any intraperitoneal surgery can lead to late complications such as small bowel obstruction from adhesions following surgery. Thus, our assumption that patients following appendectomy will return to the general population’s QALYs and mortality rate is not necessarily an accurate reflection of all clinical courses. However, the overwhelming majority of appendectomy patients recover fully post-surgery and we do not believe the above complications would significantly change our analysis. We also assumed that all patients with recurrent appendicitis following medical management would undergo surgery. However, patients who underwent nonoperative management at initial appendicitis may be more likely to be ineligible for surgery or refuse surgery during this second case of appendicitis. In addition, data were sparse for QALYs for the complications of open and laparoscopic surgery. We estimated these numbers from the EQ-5D, which while perhaps not accurate, we believe to be the best approximation given the available data. The next steps in evaluating the use of nonoperative management in uncomplicated appendicitis would be to validate the use of nonoperative management in elderly populations and to develop more accurate diagnostic criteria for uncomplicated Versus complicated appendicitis. Additionally, with increasing attention on antibiotic-resistant micro-organisms, policy decisions on the use of nonoperative management must also consider antibiotic stewardship. While one dose of perioperative antibiotics is indicated for appendectomy, treatment strategies from trial protocols for antibiotic-only management require significantly more antibiotics—some protocols require 1–3 days of IV antibiotics followed by up to 10 days of oral antibiotics. This study provides a cost-effectiveness analysis of treatment options for acute uncomplicated appendicitis among varying age groups. Our analysis demonstrates the benefit of antibiotics for initial therapy in the management of acute uncomplicated appendicitis. While the historic gold standard of laparotomy still is present as the first line treatment option in many physicians’ minds, new evidence indicates that the advancement of other methods, whether surgical via laparoscopic removal of the appendix or medical via improved antibiotic regimens, suggests better alternatives exist. Our study builds upon a growing body of literature supporting initial treatment of acute uncomplicated appendicitis with antibiotics, before surgical intervention.

